# Prey use by dingoes in a contested landscape: Ecosystem service provider or biodiversity threat?

**DOI:** 10.1002/ece3.3345

**Published:** 2017-09-21

**Authors:** Damian S. Morrant, Christopher M. Wurster, Christopher N. Johnson, James R. A. Butler, Bradley C. Congdon

**Affiliations:** ^1^ Centre for Tropical Environmental and Sustainability Science (TESS) James Cook University Cairns QLD Australia; ^2^ College of Science and Engineering James Cook University Cairns QLD Australia; ^3^ School of Biological Sciences University of Tasmania Hobart TAS Australia; ^4^ Adaptive Social and Economic Systems Program CSIRO Land and Water Flagship Brisbane QLD Australia

**Keywords:** anthropogenic, Bayesian mixing model, *Canis lupus dingo*, carnivore, conservation, diet, habitat use, predator, rainforest, stable isotope

## Abstract

In Australia, dingoes (*Canis lupus dingo*) have been implicated in the decline and extinction of a number of vertebrate species. The lowland Wet Tropics of Queensland, Australia is a biologically rich area with many species of rainforest‐restricted vertebrates that could be threatened by dingoes; however, the ecological impacts of dingoes in this region are poorly understood. We determined the potential threat posed by dingoes to native vertebrates in the lowland Wet Tropics using dingo scat/stomach content and stable isotope analyses of hair from dingoes and potential prey species. Common mammals dominated dingo diets. We found no evidence of predation on threatened taxa or rainforest specialists within our study areas. The most significant prey species were northern brown bandicoots (*Isoodon macrourus*), canefield rats (*Rattus sordidus*), and agile wallabies (*Macropus agilis*). All are common species associated with relatively open grass/woodland habitats. Stable isotope analysis suggested that prey species sourced their nutrients primarily from open habitats and that prey choice, as identified by scat/stomach analysis alone, was a poor indicator of primary foraging habitats. In general, we find that prey use by dingoes in the lowland Wet Tropics does not pose a major threat to native and/or threatened fauna, including rainforest specialists. In fact, our results suggest that dingo predation on “pest” species may represent an important ecological service that outweighs potential biodiversity threats. A more targeted approach to managing wild canids is needed if the ecosystem services they provide in these contested landscapes are to be maintained, while simultaneously avoiding negative conservation or economic impacts.

## 1 INTRODUCTION

Top predators affect the distribution and abundance of animals and plants at many trophic levels (Ripple, Beschta, Fortin, & Robbins, 2014; Schmitz, Hambäck, & Beckerman, 2000). These effects are often intensified in human‐modified landscapes where anthropogenic subsidies allow predators to reach densities that cannot be sustained by wild prey alone (Chavez & Gese, 2006; Rodewald, Kearns, & Shustack, 2011). This can result in spillover predation on native species inhabiting adjacent natural areas. Consequently, many large predators in human‐modified landscapes are believed to threaten biodiversity (Fritts, Stephenson, Hayes, & Boitani, 2003; Sillero‐Zubiri, Hoffmann, & Macdonald, 2004; Treves, Wallace, Naughton‐Treves, & Morales, 2006).

Alternatively, in Australia and elsewhere, top predators can play an important role in limiting populations of native and exotic agricultural pests (Allen, 2015; Ritchie et al., 2012). Predation alone, or in tandem with pest control, can hold pest populations below levels at which impacts are significant (Burnett, 1995; Ritchie et al., 2012; Saunders, Peisley, Rader, & Luck, 2016) and where this occurs reduction in top predators can lead to significant increases in crop and pasture losses.

Human impacts can negatively affect predators by impeding movement and habitat/prey use, while also increasing mortality due to persecution (Sillero‐Zubiri et al., 2004). Therefore, understanding predator‐prey interactions in peri‐urban and agricultural systems is essential for the development of management strategies that allow the coexistence of biodiversity and predators (Baker, Boitani, Harris, Saunders, & White, 2008; Campos, Esteves, Ferraz, Crawshaw, & Verdade, 2007; Lavin, van Deelen, Brown, Warner, & Ambrose, 2003), while simultaneously maximizing the ecosystem services predators provide.

**Figure 1:**
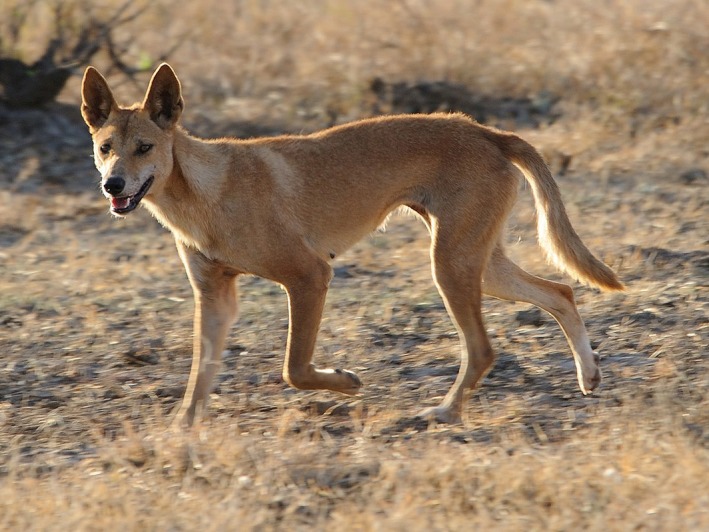
Adult female dingo, *Canis lupus dingo*

Dingoes (*Canis lupus dingo*; Figure 1) are the top predators in most Australian terrestrial ecosystems (Butler et al., 2014; Corbett, 2001; Davis et al., 2015). They prey on a broad range of taxa with population densities and diets varying in response to habitat and prey availability (Brook & Kutt, 2011; Corbett, 2001). In areas where they are artificially supplemented, dingoes can occur at high densities (Fleming, Allen, & Ballard, 2012; Newsome, Ballard, Dickman, Fleming, & Howden, 2013) and their broad hunting niche means that they have the potential to reduce prey biodiversity (Allen et al., 2016; Letnic, Ritchie, & Dickman, 2011; Ritchie et al., 2012). Consequently, dingoes have been implicated in the declines of a number of native Australian species (Allen & Fleming, 2012; Corbett, 2001).

In the Australian Wet Tropics, dingo populations may be subsidized by anthropogenic food resources and abundant generalist prey. In this same region, dingoes are considered a potential threat to native forest species (Congdon & Harrison, 2008). The highest vertebrate species diversity in the region occurs in sclerophyll habitats. However, regional endemism is much higher in rainforest (25%; Williams, Pearson, & Walsh, 1996), suggesting that at the species level, it is rainforest endemics that may be most threatened by dingo predation. One justification for unrestricted lethal control of dingoes in the Wet Tropics, and elsewhere, is this perceived threat to native fauna. However, evidence of impacts on native species is equivocal, firstly because of an inability to discriminate between attacks by dingoes and domestic/feral dogs (Congdon & Harrison, 2008) and secondly because during high activity and rapid movement dingoes of the lowland Wet Tropics use open/sugarcane habitats where common, generalist prey are abundant; rainforests are rarely used (Morrant, Johnson, Butler, & Congdon, 2017).

The effective management of predators in peri‐urban and agricultural systems requires an understanding of their prey use relative to ecological context (Bacon, Becic, Epp, & Boyce, 2011). To date, most studies of dingo diet have analyzed prey remains in scats and stomach contents (Brook & Kutt, 2011; Corbett, 2001; Vernes, Dennis, & Winter, 2001). However, such methods can significantly under‐represent specific prey types, provide only a snapshot of a predator's diet, and are affected by the size and digestibility of prey items (Milakovic & Parker, 2011; Roth & Hobson, 2000). Recent advances in stable isotope analysis offer advantages over traditional diet analysis, as it provides information about prey types integrated over time and space (Layman et al., 2012). By measuring the stable isotope composition (e.g., δ^13^C and δ^15^N values) in tissues of consumers, it is possible to determine the stable isotope composition of prey and so infer the principal habitats from which prey are sourced (Crawford, McDonald, & Bearhop, 2008; Wurster et al., 2012).

In this study, we aimed to establish the potential threat posed by dingoes to native fauna in the lowland Wet Tropics. To do this, we used conventional diet analysis to determine the major prey items used by dingoes and to test whether threatened taxa were consumed. We also used stable isotope analysis of dingo and prey hair to identify the potential source habitats for prey. This allowed us to determine the extent to which dingoes source prey from rainforest habitats where threatened taxa are most likely to be encountered.

## 2 MATERIALS AND METHODS

### 2.1 Study area

**Figure 2:**
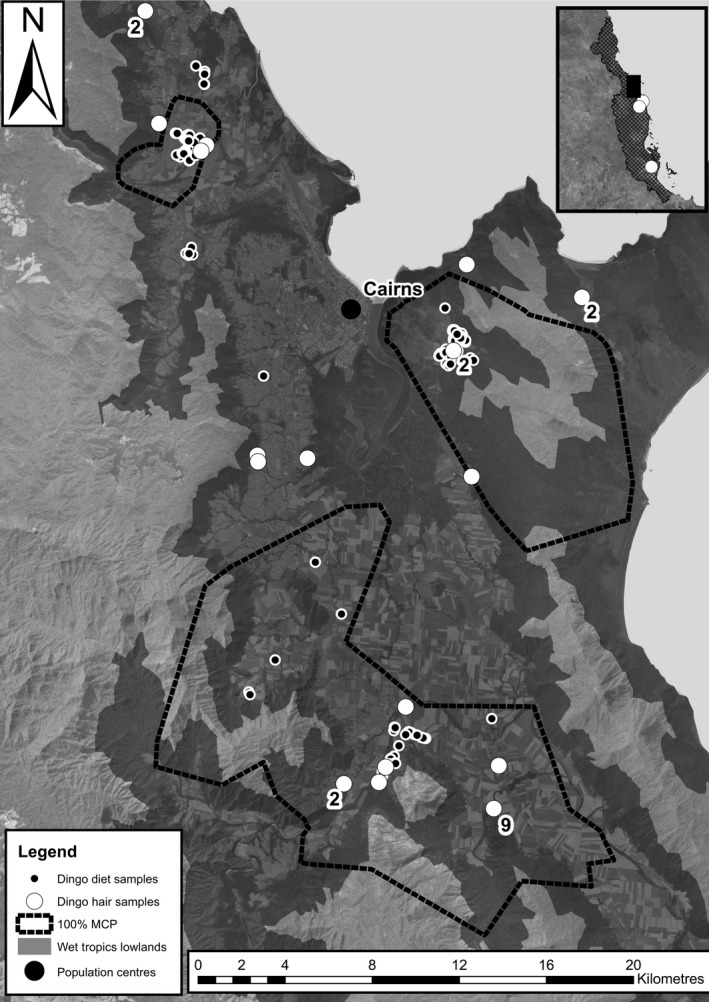
Locations from which dingo hair and diet samples were collected. Numbers next to hair samples represent >1 individual. The combined home range boundaries of nine dingoes, GPS tracked during a concurrent study (Morrant et al., 2017), are indicated by 100% MCP. The inset shows Wet Tropics Bioregion (hatched), area of main map (black rectangle), and three additional locations from which hair samples were collected (white circles)

The study was conducted in the lowland Wet Tropics of northeastern Australia between 16°48′S, 145°41′E and 17°24′S, 145°55′E (Figure 2). The vegetation is a mosaic of tropical rainforests, open wet sclerophyll forests, sedgeland, and grassland, adjacent to large areas of sugarcane, urban development and cattle pasture. Dingoes in the region have access to a range of native and feral animal prey and anthropogenic food resources including human refuse and human‐killed feral pig carcasses (Morrant et al., 2017).

### 2.2 Sample collection

Between 2009 and 2011, we analyzed dingo scats collected opportunistically, and stomach contents of dingoes killed during pest control (carcasses; Figure 2). Between August 2007 and 2012, we also collected hair and vibrissae from wild adult dingoes (34 hair; 14 vibrissae), from four sources: (1) hair traps; (2) carcasses of dingoes killed during pest control; (3) road kills; and (4) ear notches from live captures. We collected hair opportunistically from potential prey species (prey hair) in the same region between 2012 and 2014, from specimens trapped by other researchers and from animals killed by vehicle strike. All hair samples were stored at −18°C.

### 2.3 Sample analysis

#### 2.3.1 Diet from scats and stomach contents

Animal remains in stomach contents and scats were identified from hair structure, skin, feathers, invertebrate exoskeletons, and bones (Georgeanna Story; Scats About, Majors Creek, NSW). Prey composition was recorded as frequency of occurrence for each prey species (the number of occurrences of each prey species divided by the total number of scats and/or stomachs; Corbett, 1989; Brook & Kutt, 2011).

#### 2.3.2 Stable isotopes in body hair and vibrissae

Samples were prepared for stable isotope analysis using a modified version of the methods of Wurster et al. (2012). Samples were agitated in 2:1 (v/v) dichloromethane:methanol for 15 min to remove surface debris and oils (washing), or wiped clean, and then air‐dried at room temperature for 24 hr. A subset of dingo hair samples that had been stored in ethanol was also washed and freeze‐dried for 24 hr. All hair samples were then crushed and homogenized for 3 min in a Wig‐L‐Bug grinder (Crescent Dental Co., Chicago, Ill.). Samples of ~0.1 mg were then loaded into tin capsules and crimped for stable carbon and nitrogen isotope composition and elemental abundance via elemental analysis isotope ratio mass spectrometry (EA‐IRMS).

Carbon and nitrogen stable isotope ratios were measured on a Costech 4010 Elemental Analyzer fitted with a zero‐blank auto‐sampler coupled via a ConFloIV to a Thermo Scientific DeltaV^PLUS^ using continuous‐flow isotope ratio mass spectrometry (EA‐IRMS). Stable isotope ratios are reported as per mil (‰) deviations from the VPDB and AIR reference standard scale for δ^13^C and δ^15^N values, respectively. Precisions (*SD*) on internal standards were better than ±0.1‰ and 0.2‰ for carbon and nitrogen, respectively. USGS‐40 and two internal standards (*Oxyuranus scutellatus* keratin [taipan snake; collected in sugarcane], and chitin) were analyzed with samples and used for calibration.

#### 2.3.3 Estimation of habitat use from stable isotopes

We investigated resource and habitat use of dingoes by comparing the isotope values (δ^13^C and δ^15^N) in dingo hair with values obtained from prey hair. Previously, we had observed that open sugarcane/grassland habitats dominated by C_4_ vegetation are the most important habitats for lowland Wet Tropics dingoes during periods of high activity (Morrant et al., 2017). Therefore, we analyzed to determine whether prey were more likely to have originated in open habitats, independent of prey species.

To undertake this analysis, we grouped the isotope values of all prey (converted from hair to muscle values; described below), regardless of taxonomic status, into three categories, according to their δ^13^C values, using a K‐means cluster analysis (SPSS Statistics for Windows; IBM Corp., Armonck, NY) with three forced means representing: (1) rainforest dwellers (forest); (2) animals which move between habitats, or live in open woodlands or on open rainforest ecotones (mixed); and (3) open grassland/sugarcane dwellers (open). Woody vegetation, such as rainforest, predominantly employs a C_3_ photosynthesis, whereas grasses in this region (including sugarcane) predominantly use C_4_ photosynthesis (Wurster et al., 2012). Therefore, these habitat categories were intended to provide a spectrum that could be used to determine whether dingoes or prey primarily sourced their nutrients from either C_3_ or C_4_ vegetation types. We used a K nearest‐neighbor randomization test to determine whether stable isotope ratios were significantly different among the three habitat groups, and therefore appropriate for analysis with *siar* (Rosing, Ben‐David, & Barry, 1998).

We converted dingo hair/vibrissae δ^13^C and δ^15^N values to diet equivalents, using +4.3‰ and +3.1‰ as our dingo hair–diet and vibrissae–diet discrimination values, for δ^13^C and δ^15^N, respectively, as per those obtained for captive gray wolves (*Canis lupus*) (sensu McLaren, Crawshaw, & Patterson, 2015).We converted prey hair δ^13^C values to muscle equivalents, because dingoes’ nutrient intake from ingesting prey is primarily derived from flesh. Discrimination values were not available for most prey species. Therefore, we used average values for mammalian herbivores as approximate prey hair–muscle discrimination values for δ^13^C and δ^15^N (+3.2‰ and +2.5‰ for δ^13^C and δ^15^N, respectively) (Sponheimer, Robinson, Ayliffe, Passey, et al., 2003; Sponheimer, Robinson, Ayliffe, Roeder, et al., 2003). We acknowledge that using average prey discrimination values adds uncertainty that could be avoided if species‐specific values could be applied. However, our requirement for *siar* analysis was only that a range of prey values were available, along a continuum from an exclusively C_4_ diet to exclusively C_3_ diet. Consequently, we used the green ringtail possum (*Pseudochirops archeri*) which is an obligate rainforest folivore (Winter, Krockenberger, & Moore, 2008), to establish the rainforest endpoint of this continuum and to validate that our transformed values matched previously published isotopic data on rainforest specialists (Wurster et al., 2012). Finally, we used the *siar* package in R (Parnell, Inger, Bearhop, & Jackson, 2008) to generate a Bayesian mixing model estimate of the proportion of prey items sourced by dingoes from each of the different habitat categories.

We investigated temporal changes in resource and habitat use of dingoes by comparing the isotope values (δ^13^C) in individual vibrissae over time using a general linear mixed model with individual as a random factor in the *lme4* package in R (Bates et al., 2017). This analysis accounted for the variation between individuals but did not test for individual differences. Observations in wolves suggest that growth rates in *Canis* spp. vibrissae are seasonally variable and may vary within and among individuals (McLaren et al., 2015). Therefore, we did not ascribe any seasonality to our measurements. Our primary aim was to determine whether, in general, dingoes’ use of habitat changed over time.

## 3 RESULTS

### 3.1 Dietary determination from scats and stomach contents

We recorded 27 different food types in 259 fecal and 10 stomach samples. Almost all samples (96%) contained the hair, bones, or teeth of mammals (Table 1). Most (66.5%) contained only one discernible prey species, 25.7% contained two, 4.5% contained three, and one scat contained four species. Birds were found in 24 (9%) of scats but constituted 100% of the sample in only one; no scats contained cassowary remains. Five samples contained beetles, three skinks, two grasshoppers, two fish, and one each contained frog, bluetongue lizard (*Tiliqua scincoides*), and unidentified reptile eggs. Three percent of samples contained only vegetation, primarily grass, but also fruit, and one contained sugarcane. Four samples contained plastic, three string, and one paper. Nonmammalian prey species were excluded from further analyses because they composed a relatively minor component of dingo diet. The five most commonly recorded prey species were native mammals. However, no rare or threatened mammals were recorded in any sample. Introduced mammal species composed <1% of species recorded, and of these feral pigs were the most common prey.

**Table 1:** Mammalian species in 269 dingo diet samples from the lowland Wet Tropics of Australia, collected between 2010 and 2012

Common name	Species	Frequency	
		*n*	Rank
Northern brown bandicoot	*Isoodon macrourus*	111	1
Canefield rat	*Rattus sordidus*	63	2
Agile wallaby	*Macropus agilis*	45	3
Fawn‐footed melomys	*Melomys cervinipes*	21	4
Grassland melomys	*Melomys burtoni*	19	5
Unidentified rat	*Rattus* sp.	15	6
Feral pig	*Sus scrofa*	14	7
Red‐legged pademelon	*Thylogale stigmatica*	4	8
Unidentified macropod	*Macropus* sp.	3	9.5
Black rat	*Rattus rattus*	3	9.5
Eastern gray kangaroo	*Macropus giganteus*	2	11.5
Swamp wallaby	*Wallabia bicolor*	2	11.5
Common brushtail possum	*Trichosurus vulpecula*	1	13
Greater glider	*Petauroides volans*	1	13
Echidna	*Tachyglossus aculeatus*	1	13
Cape York rat	*Rattus leucopus*	1	13
Bush rat	*Rattus fuscipes*	1	13
Giant white‐tailed rat	*Uromys caudimaculatus*	1	13
Domestic bovine	*Bos taurus/Bos indicus*	1	13
Goat	*Capra hircus*	1	13
Total			

### 3.2 Stable isotopes in prey hair

**Figure 3:**
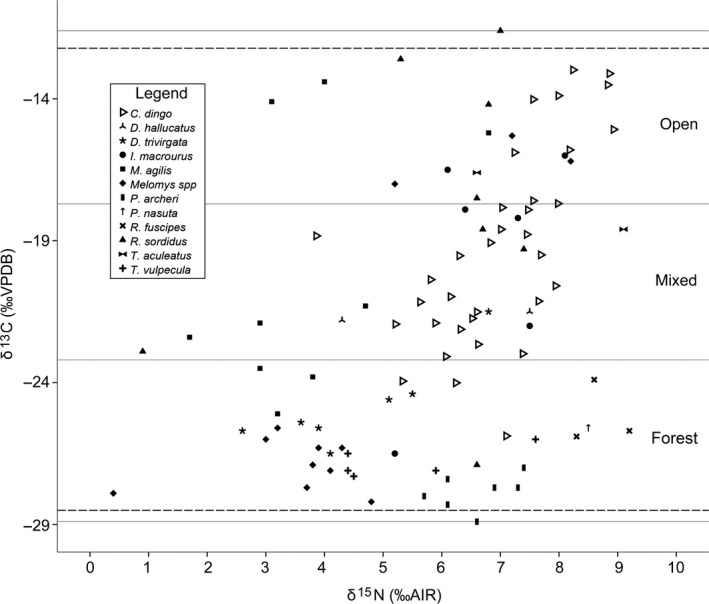
Isotope values in the hair of dingoes (*n* = 34) and potential prey species in the lowland Wet Tropics. Hair‐to‐diet discrimination values have been added to dingoes (+4.3‰ and +3.1‰ for δ^13^C and δ^15^N, respectively; McLaren et al., 2015) and prey (+3.2‰ and +2.5‰ for δ^13^C and δ^15^N, respectively; sensu Sponheimer, Robinson, Ayliffe, Passey, et al., 2003; Sponheimer, Robinson, Ayliffe, Roeder, et al., 2003). The figure is divided into the three habitat categories, determined using a K‐Means Cluster Analysis (open, mixed, and forest). Dashed horizontal lines show entirely C_4_ plant‐based diet (top) and entirely C_3_ plant‐based diet (bottom) endmembers sensu Wurster et al. (2012)

We analyzed 62 hair samples from 11 potential prey species (Figure 3, Table 2). All isotopic values discussed below are the original hair values unless otherwise indicated (i.e., discrimination values have not been applied). The isotopic values in hair of all potential prey species sampled, including species not identified in diet samples, had a broad range of δ^13^C and δ^15^N values (Figure 3, Table 2). Of the three most frequently occurring prey taxa identified in the diet analysis, *Rattus sordidus* δ^13^C values ranged from −8.4‰ to −23.7‰, *Macropus agilis* from −10.2‰ to −21.9‰, and *Isoodon macrourus* from −12.8‰ to −23.3‰. Conversely, the δ^13^C values of the green ringtail possums, which are rainforest specialists and are not known prey of dingoes, showed little variability (−23.8‰ to −25.7‰).

**Table 2:** Stable isotope values (δ^13^C and δ^15^N) in hair of potential dingo prey from the Wet Tropics of Australia. Discrimination values have not been applied

Common name	Species	*n*	$\overset{¯}{X}$ δ^13^C (±*SE*)	$\overset{¯}{X}$ δ^15^N (±*SE*)
Echidna	*Tachyglossus aculeatus*	2	−14.4 (±0.47)	10.1 (±0.59)
Canefield rat	*Rattus sordidus*	8	−14.7 (±1.85)	8.2 (±0.74)
Northern brown bandicoot	*Isoodon macrourus*	6	−16.3 (±1.64)	9.1 (±0.43)
Agile wallaby	*Macropus agilis*	9	−16.9 (±1.51)	6.0 (±0.48)
Northern quoll	*Dasyurus hallucatus*	2	−18.5 (±0.07)	8.2 (±0.75)
Grassland/fawn‐footed melomys	*Melomys burtoni/cervinipes*	12	−21.0 (±1.86)	6.6 (±0.75)
Striped possum	*Dactylopsila trivirgata*	7	−21.6 (±0.61)	6.8 (±0.53)
Bush rat	*Rattus fuscipes*	3	−22.0 (±0.37)	11.0 (±0.15)
Long‐nosed bandicoot	*Perameles nasuta*	1	−22.4	10.8
Brushtail possum	*Trichosurus vulpecula*	5	−23.6 (±0.18)	7.6 (±0.47)
Green ringtail possum	*Pseudochirops archeri*	7	−24.7 (±0.24)	8.9 (±0.25)

### 3.3 Stable isotopes in dingo hair

**Figure 4:**
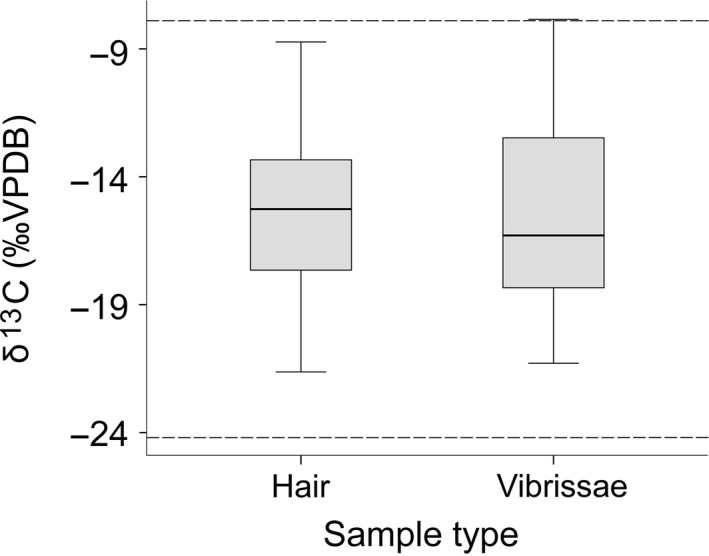
Mean δ13C values of dingo hair (*n* = 34) and segments from 14 dingo vibrissae (*n* = 158). Discrimination values have not been applied. Entirely C4 plant‐based diet (top) and entirely C3 plant‐based diet (bottom) endmembers (dashed horizontal lines; sensu Wurster et al. 2012) have been adjusted by +4.3‰ to account for hair–diet discrimination (sensu McLaren et al., 2015)

δ^13^C and δ^15^N values were measured for 34 individual dingo hair samples. δ^13^C values ranged from −8.7‰ to −21.6‰ (mean −15 ± *SE* 0.59; Figure 4). δ^15^N values ranged between 7‰ and 12‰ (mean 10.1 ± *SE* 0.19). Sex data were recorded for 28 animals (13M; 15F) but were not available for six ear samples provided by pest managers. There was no significant difference between male and female δ^13^C values (male mean −15.23‰ ± *SE* 0.69, female mean −14.45‰ ± *SE* 1.03; independent‐samples *t* tests, *t* (24) = 0.637, *p* = .53, two‐tailed), or δ^15^N values (male mean 9.9‰ ± *SE* 0.34, female mean 10.4‰ ± *SE* 0.25; *t* (23) = 1.20, *p* = .24, two‐tailed).

### 3.4 Siar modeling—habitat categories as dietary source

**Figure 5:**
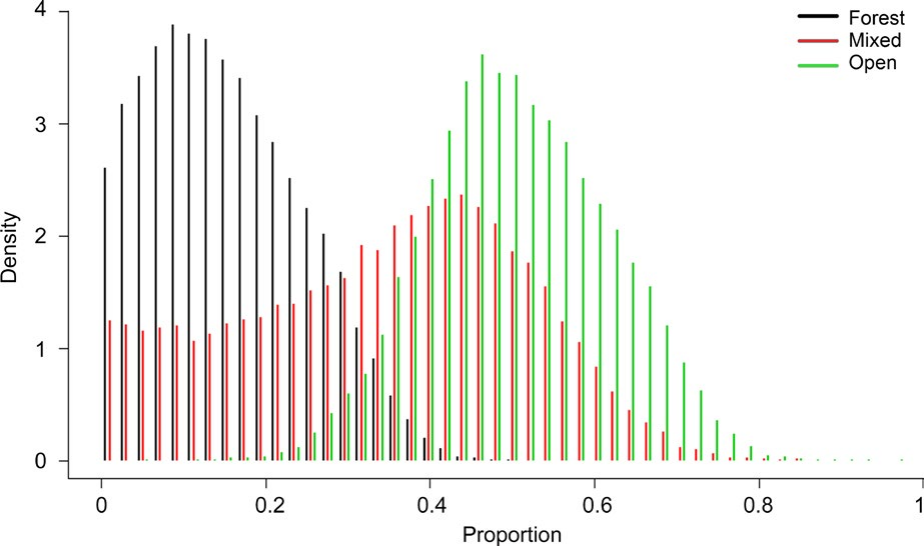
Relative contribution of dingo diet components according to Bayesian mixing models, where prey were grouped into three categories along a gradient from a primarily C_3_ diet (forest), to a mix of C_3_ and C_4_ (mixed), to a primarily C_4_ diet (open). Discrimination values were applied to dingo hair to convert to diet values (+4.3‰ and +3.1‰ for δ^13^C and δ^15^N, respectively; sensu McLaren et al., 2015), and prey hair to convert to muscle values (+3.2‰ and +2.5‰ for δ^13^C and δ^15^N, respectively; sensu Sponheimer, Robinson, Ayliffe, Passey, et al., 2003; Sponheimer, Robinson, Ayliffe, Roeder, et al., 2003)

Prey δ^13^C (‰) values (with discrimination values applied sensu Sponheimer, Robinson, Ayliffe, Passey, et al., 2003; Sponheimer, Robinson, Ayliffe, Roeder, et al., 2003) occurred within all three habitat categories representing a continuum from a primarily C_4_ diet (open; −10.0‰ to −16.3‰ δ^13^C), through a mixed C_3_/C_4_ diet (mixed; −16.6‰ to −21.9‰ δ^13^C), to a primarily C_3_ diet (forest; −22.2‰ to −27.3‰ δ^13^C). Prey values were variable (χ^2^_(10)_ = 34.542, *p* = <.05), indicating that most prey sourced nutrients from multiple habitats and that habitat use differed among individuals, independent of prey species (Figure 3, Table 2). Isotope values from 30 dingoes were used in the *siar* model; four animals for which we did not have exact location data were excluded. The estimated relative contribution of prey originating in each habitat type was (Figure 5) as follows: forest (low–high 95% hdr: 0–32%; mode 18%), mixed (low–high 95% hdr: 0–61%; mode 43%), and open (low–high 95% hdr: 29–72%; mode 46%).

### 3.5 Stable isotopes in vibrissae—temporal variation in dietary sources

There was no significant difference between the mean δ^13^C values in vibrissae and body hair samples, for 12 dingoes from which we analyzed both hair and vibrissae (*t*_[11]_ = −1.47, *p* = .17, Figure 4). Sequential δ^13^C values for 14 individual dingo vibrissae varied similar to body hairs and ranged from −7.9 to −21.3‰; δ^15^N values ranged from 4.1‰ to 9.5‰. When individual variation was accounted for, there was no significant difference in stable isotope values between vibrissae segments over time (*lme4*: χ^2^_(1)_ = 3.42, *p* = .064). Thus suggesting that in general individual dingoes did not markedly change prey types or patterns of habitat use over time.

## 4 DISCUSSION

Dingoes in the lowland Wet Tropics prey primarily on common native mammals in open and mixed habitats. The most frequently recorded prey were northern brown bandicoots, canefield rats, agile wallabies, and *Melomys* species, all of which are abundant in open habitats of the region. This finding is partially consistent with a previous study that identified northern brown bandicoots and agile wallabies as important dietary components in peri‐urban “wild dogs” of “North Queensland”. However, previously canefield rats and *Melomys* species were either not recorded, or occurred as negligible dietary components (Allen et al., 2016), these differences likely reflecting the small number of “North Queensland” scats collected in sugarcane croplands during that study (Allen et al. 2016).

Feral pigs comprised a relatively large portion of dingo diet in the current study, a finding also observed in the upland Wet Tropics (Burnett, 1995) and elsewhere in Australia (Corbett, 1989, 1995; Newsome, 1990). We observed a number of instances of consumption of pigs during concurrent radio‐tracking and camera‐trapping studies, and while most were of dingoes scavenging carcasses, adult dingoes also preyed on live pigs (Morrant, 2015).

Anthropogenic food sources were uncommon in diet samples and did not appear to constitute an important dietary component. This finding is further supported by tracking studies in the lowland Wet Tropics showing that dingoes generally do not frequent locations where anthropogenic food is available, apart from visiting sites where landholders dispose of pig carcasses (Morrant et al., 2017). However, as food scraps may be completely digested (Allen et al., 2016), it is difficult to discount the use of artificial food sources based on scats alone.

The stable isotope composition of potential prey differed by more than 15‰, and even within individual species, the variation was sometimes considerable. The patchy nature of agricultural landscapes in the lowland Wet Tropics means that prey feeding on both C_3_ and C_4_ vegetation types are found in sugarcane croplands. Similarly, C_3_ and C_4_ resources are available in open sclerophyll forests and woodlands with grassy ground layers. Conversely, carbon isotope availability in rainforests landscapes tends to be more homogenously C_3_ (Wurster et al., 2012). If dingoes source their prey primarily in rainforests, we expect their δ^13^C values to be light (i.e., ~−28.5‰), whereas if they take prey from open forest/woodland or sugarcane habitats their δ^13^C values would be heavier and more variable (≤−12.2‰). For example, δ^13^C values of green ringtail possums, which are rainforest specialists (Winter et al., 2008), showed little variability, whereas the δ^13^C values of agile wallabies, which use forest/woodland edges during the day and forage in open habitats at dawn and dusk (Stirrat, 2004), were much more variable. Consequently, unless dingoes prey exclusively on rainforest taxa, knowledge of the δ^13^C values of prey species alone is an unreliable indicator of land use in the lowland Wet Tropics.

Previous research has demonstrated that δ^15^N values are variable in the Wet Tropics (Wurster et al., 2012), which may be largely attributable to the use of nitrogen‐rich fertilizers in sugarcane agriculture (Ostrom, Hedin, von Fischer, & Robertson, 2002; Wurster et al., 2012). We found considerable variation in δ^15^N values within dingoes and prey. Consequently, δ^15^N values could not be used to estimate the principal trophic level at which dingoes sourced prey.

Our Bayesian mixing modeling suggested that dingoes in the lowland Wet Tropics source prey across a range of habitats, from closed rainforest to open grassland/sugarcane; however, prey from the open and mixed categories represented the vast majority of prey ingested. Prey from the open habitat category were the greatest contributor to dingo diet, followed closely by prey from the mixed category. Prey from the forest category provided only a relatively small contribution to dingo diet. The C_4_ in dingo diets is unlikely to have come from rainforest specialist prey, or generalist prey feeding in rainforest habitats. This is because C_4_ vegetation is generally unavailable in rainforests. Therefore, the majority of C_4_ in dingo diets is attributable to prey from open woodland/grassland and sugarcane habitats. Alternatively, as discussed above, not all C_3_ in dingo diets will be derived from rainforest specialists, or from prey taken in rainforest habitats. Some native prey, particularly agile wallabies, feed in a range of habitat types including rainforest, and tracking studies (Morrant et al., 2017) clearly suggest that such prey are more likely to be taken by dingoes in open habitats.

When individual variation among vibrissae was accounted for, there was no significant difference in stable isotope values across time within individuals. Therefore, dingoes in the lowland Wet Tropics appear to prey on a broad range of taxa with each animal consistently using a specific range of prey and/or habitat types (sensu Bearhop, Adams, Waldron, Fuller, & Macleod, 2004's Type B generalist).

Elsewhere, it has been suggested that predation by dingoes may be an important factor modifying or limiting populations of agricultural “pest” species (Allen, 2015; Glen, Dickman, Soulé, & Mackey, 2007; Letnic et al., 2011). While exotic animals are the most commonly recognized “pest” taxa in most agricultural systems, native fauna can also be considered as “pests” where their activity leads to financial losses (Dyer, Clarke, & Fuller, 2011; Glen et al., 2007; Hunt, Dyer, Kerkwyk, Marohasy, & Thompson, 2004; Letnic et al., 2011). Rodents, both native and exotic, are significant “pests” of sugarcane in North Queensland and cause serious damage to crops, particularly during population outbreaks (Dyer et al., 2011; Hunt et al., 2004). Our results suggest that by taking advantage of high abundances of “pest” species in open grassland and sugarcane landscapes, the dingoes in our study and elsewhere in the region likely provide an important ecosystem service to sugarcane producers.

We found no evidence of threatened species in dingo diets, or that individual dingoes hunt exclusively in rainforests targeting rainforest specialist prey. Concurrent GPS tracking also suggests that dingoes do not hunt in rainforest where many threatened endemic species occur (Morrant et al., 2017). However, stable isotope analysis suggests that dingoes may source some of their prey from species feeding in rainforest environments. Whether these prey are actually caught in rainforests is unknown, making it impossible to rule out some level of predation on rainforest dwelling threatened species.

Previous work has suggested that dingoes may threaten “seemingly unsusceptible” species when alternative, preferred prey resources become unavailable (Allen et al., 2013; Corbett, 2001; Corbett & Newsome, 1987). However, the examples cited often relate to unusual circumstances where threatened prey are at high densities after reintroductions, or where prey diversity is low, for example on islands. Such situations are unlikely to occur in the Wet Tropics, which is highly productive and has a diverse assemblage of common, potential prey. However, some scenarios, such as a collapse of the sugarcane industry, rapid urban expansion into sugarcane habitats, or a disease outbreak that results in extensive mortality among common mammalian taxa, could render abundant prey unavailable. Under such scenarios, it is possible that dingo predation could put populations of threatened fauna at risk.

## 5 CONCLUSION

Our analysis of dingo scats and stomach contents combined with Bayesian mixing modeling suggests that dingoes in the lowland Wet Tropics primarily prey on common mammal species in open and mixed habitats. Although dingoes have the potential to negatively impact populations of threatened fauna, we found little evidence of them preying on threatened species in the lowland Wet Tropics, or that individual dingoes hunt primarily in rainforest. The observed preferences for common prey sourced primarily from relatively open habitats suggest that dingoes more likely provide an ecosystem service by reducing populations of agricultural pests. A similar role in reducing populations of pest species to the benefit of crop and livestock producers has been proposed for canids in anthropogenic landscapes elsewhere, including golden jackals (Jaeger, Haque, Sultana, & Bruggers, 2007), coyotes (Jones, Michael, Lashley, & Jackson, 2016), and dingoes (Allen, 2015). Future research should quantify the value of canids and other predators to primary producers to enable coexistence.

The situation in the lowland Wet Tropics presented an opportunity to investigate circumstances where anthropogenic modifications to the landscape could be expected to sustain dingo numbers at levels that would pose a threat to native fauna. Therefore, the lowland Wet Tropics provided a model system for understanding the potential ecological impacts of dingoes or other wild canid predators in contested landscapes in general. Current broad‐spectrum dingo control strategies in the region, and likely elsewhere, appear to be a disproportionate one‐size‐fits‐all response to minimizing principally the perceived threats to livestock and secondarily potential threats to native species, where much of the evidence for native species impact remains anecdotal. This is also likely the situation in many other systems where wild canids are subject to unregulated lethal control.

It should be noted that details of hunting patterns and prey use suggest that under specific conditions, or in particular environmental contexts, dingoes could preferentially target native prey of conservation concern. However, it seems that dingoes, and *Canis* spp. in general, pose little threat to rainforest specialists. Therefore, our findings suggest that a more targeted, location or pack specific, management approach is needed if the ecosystem services provided by dingoes are to be maintained while simultaneously avoiding either conservation or economic impacts.

## CONFLICT OF INTEREST

None declared.

## AUTHORS’ CONTRIBUTIONS

DM, BC, CJ, and JB conceived the ideas and designed methodology; DM collected the data; DM and BC analyzed the data; DM and CW conducted stable isotope analysis; DM and BC led the writing of the manuscript. All authors contributed critically to the drafts and gave final approval for publication.

## DATA ACCESSIBILITY

We intend to archive our data in the Dryad repository. A DOI will be provided before final acceptance of the paper for publication.
